# Proteomics study of human cord blood reticulocyte-derived exosomes

**DOI:** 10.1038/s41598-018-32386-2

**Published:** 2018-09-19

**Authors:** Míriam Díaz-Varela, Armando de Menezes-Neto, Daniel Perez-Zsolt, Ana Gámez-Valero, Joan Seguí-Barber, Nuria Izquierdo-Useros, Javier Martinez-Picado, Carmen Fernández-Becerra, Hernando A. del Portillo

**Affiliations:** 1 ISGlobal, Hospital Clínic - Universitat de Barcelona, Barcelona, Spain; 2 IrsiCaixa AIDS Research Institute, Badalona, Spain; 3 IGTP Institut d’Investigació Germans Trias i Pujol, Badalona, Spain; 4 Universitat de Vic – Universitat Central de Catalunya (UVic-UCC), Vic, Spain; 5 Institució Catalana de Recerca i Estudis Avançats (ICREA), Barcelona, Spain; 6 Present Address: Instituto Aggeu Magalhães-FIOCRUZ, Recife, Pernambuco Brazil; 7 Present Address: Department of Pathology & REMAR-IVECAT Group, Hospital Universitari and Health Sciences Research Institute Germans Trias i Pujol, Universitat Autònoma de Barcelona, Barcelona, Spain

## Abstract

Reticulocyte-derived exosomes (*Rex*), extracellular vesicles of endocytic origin, were initially discovered as a cargo-disposal mechanism of obsolete proteins in the maturation of reticulocytes into erythrocytes. In this work, we present the first mass spectrometry-based proteomics of human *Rex* (*HuRex*). *HuRex* were isolated from cultures of human reticulocyte-enriched cord blood using different culture conditions and exosome isolation methods. The newly described proteome consists of 367 proteins, most of them related to exosomes as revealed by gene ontology over-representation analysis and include multiple transporters as well as proteins involved in exosome biogenesis and erythrocytic disorders. Immunoelectron microscopy validated the presence of the transferrin receptor. Moreover, functional assays demonstrated active capture of *HuRex* by mature dendritic cells. As only seven proteins have been previously associated with *HuRex*, this resource will facilitate studies on the role of human reticulocyte-derived exosomes in normal and pathological conditions affecting erythropoiesis.

## Introduction

Research on exosomes is gaining momentum as these vesicles of endocytic origin act in intercellular communication and represent novel therapeutic strategies and non-invasive biomarkers of disease^1–3^. During their maturation to erythrocytes, reticulocytes selectively remove proteins, noticeably the transferrin receptor (TfR or CD71), as well as membrane-associated enzymes, through the formation of multivesicular bodies which after fusion with the plasma membrane release intraluminal vesicles, termed exosomes^4–6^.

Studies on the protein cargo composition of reticulocyte-derived exosomes (*Rex*) are mostly limited to animal models and validations through non high-throughput technologies^7–9^. These studies clearly established that *Rex* represent a selective cargo disposal mechanism in the terminal maturation of reticulocytes into erythrocytes^7^. More recently, mass spectrometry (MS)-based proteomic analysis of *Rex* from phenylhydrazine-treated rats^10^ and from mice infected with rodent malaria parasites with a unique tropism to reticulocytes^11^, described the proteome of *Rex* in these species. Results reinforced the view that *Rex* have selective cargo and demonstrated for the first time that *Rex* from malaria-infected reticulocytes contain parasite antigens involved in antigen presentation and capable of modulating immune responses^11,12^. To our knowledge, however, only seven proteins (TfR, Stomatin, Flotillin-1 and 2, CD55, CD58 and CD59) have been previously reported in human *Rex*^7,13,14^.

In this work, we present the first MS-based proteomic profile of human reticulocyte-derived exosomes (*HuRex*). *HuRex* were isolated from cultures of human cord blood using two different culture conditions, absence/presence of exosome-depleted serum, and two different exosome isolation techniques, size-exclusion chromatography (SEC) and ultracentrifugation (UC). The proteome consists of 367 proteins most of which have not been previously reported. Immunoelectron microscopy validated the presence of the transferrin receptor, a major *Rex* component, and comparative analysis with the MS-datasets from reticulocytes^15–17^ and mature red blood cells^17–20^, rendered a selected list of *HuRex* plasma membrane and cytosol proteins. In addition, we identified proteins involved in antigen presentation and observed an active capture of *HuRex* by mature dendritic cells. These results thus provide a first base-line proteome of *HuRex* enabling further studies not only on their biogenesis and antigen-presenting capacity, but also on their cargo composition in diseases affecting erythropoiesis.

## Results

### Enrichment of human reticulocytes and *in vitro* production and characterization of human reticulocyte-derived exosomes

Enriched reticulocyte samples were obtained from human blood of umbilical cords, a source with higher percentage of reticulocytes than adult humans’ blood and with no major proteomic differences between them^17^. After removal of leukocytes and concentration on Percoll gradients (Supplementary Fig. S1), yield percentages of reticulocytes ranged between 20 to 60% among different donors. Reticulocytes were subsequently cultured *in vitro* for 36 h at 1–3% hematocrit. Of note, our cultures contain significant percentages of mature red blood cells, yet these cells lack endocytic machinery and it has been clearly established that they do not secrete exosomes^6,21^. Furthermore, we discarded reticulocyte-enriched preparations that contained over 2% of contaminating leukocytes to minimize the presence of leukocyte-derived vesicles in the cultures. We refrained from using CD71 affinity bead purification as there is a large heterogeneous population of reticulocytes from CD71negative to CD71high^22^. *HuRex* were produced *in vitro* in the presence or absence of serum, previously depleted of exosomes, as there is evidence suggesting that the protein cargo varies significantly in the absence of serum^23^. Before and after culture, cell viability was assessed by microscopy using Trypan Blue stain. We demonstrated 95.6 ± 2.1% of cell viability after 36 hours of culture independently of serum supplementation. Thus, excluding a major proteomic confounding due to apoptotic vesicles in culture supernatants. It has also been emphasized that another factor affecting the quality of exosome preparations is the method of purification^24^; therefore, we purified *HuRex* by either UC^25^ or SEC^26^. The use of sequential centrifugation removing most apoptotic bodies (1,300 g pellet) and microvesicles (15,000 g pellet)^25^ and the use of SEC also removing these types of vesicles^26^, further emphasizes that we are mostly isolating exosomes derived from reticulocytes^27^. Due to intrinsic variability of human samples and the lack of a unique method for isolation of exosomes, however, we cannot exclude the possibility that a small number of these proteins are wrongly assigned to exosomes derived from reticulocytes.

**Figure 1:**
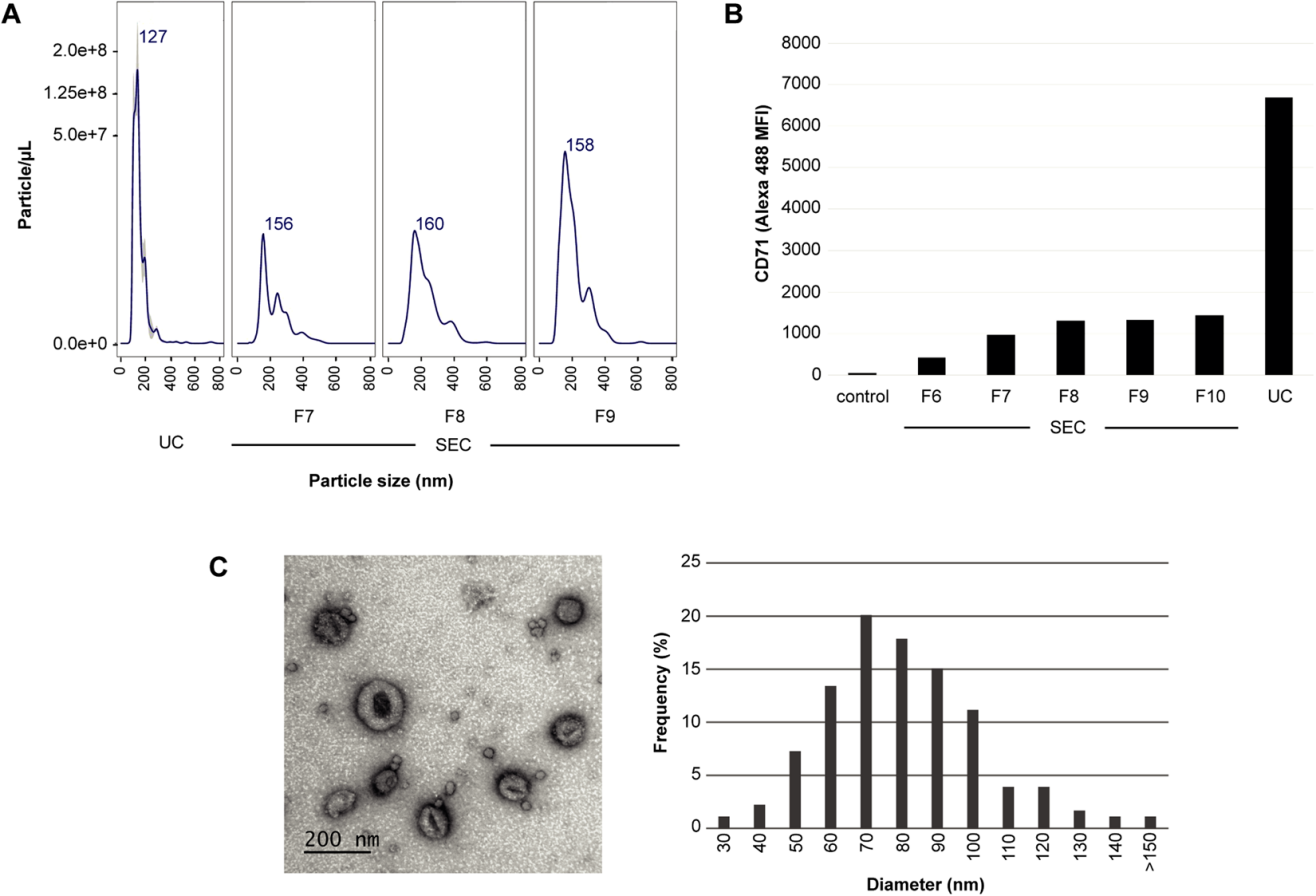
Isolation and characterization of exosomes derived from human cord blood reticulocytes. (**A**) NTA profiles of *HuRex* from ultracentrifugation (UC) and size exclusion chromatography (SEC) fractions. Concentration is shown in particle/µL. **(B)** Flow cytometry analysis of transferrin receptor, CD71, in *HuRex*. MFI, Median Fluorescence Intensity. **(C)** Electron microscopy. Representative TEM image on the left. Bar represents 200 nm. Size distribution from TEM images quantified by ImageJ on the right. nm, nanometers.

*HuRex* were first subjected to Nanoparticle Tracking Analysis (NTA). Results demonstrated that *HuRex* were homogeneous with a mean size of 127 nm and that particle concentration was in the range of 10^8^ particles/µL. Particle concentration from SEC fractions was always lower than that from UC (Fig. 1A). We determined the presence of the major component of *Rex*: the transferrin receptor, CD71^4,5,8^ by beads-based flow cytometry. Higher MFI values for UC compared to those for SEC were always observed (Fig. 1B). To confirm vesicle integrity, *HuRex* preparations obtained by UC were analyzed by transmission electron microscopy (TEM) by means of negative staining (Fig. 1C). As expected from fixation and dehydration^28^, size distribution of TEM images revealed smaller vesicle mean size (70 nm), than that recorded by NTA (Fig. 1C).

### Detection of the transferrin receptor on human reticulocyte-derived exosomes

**Figure 2:**
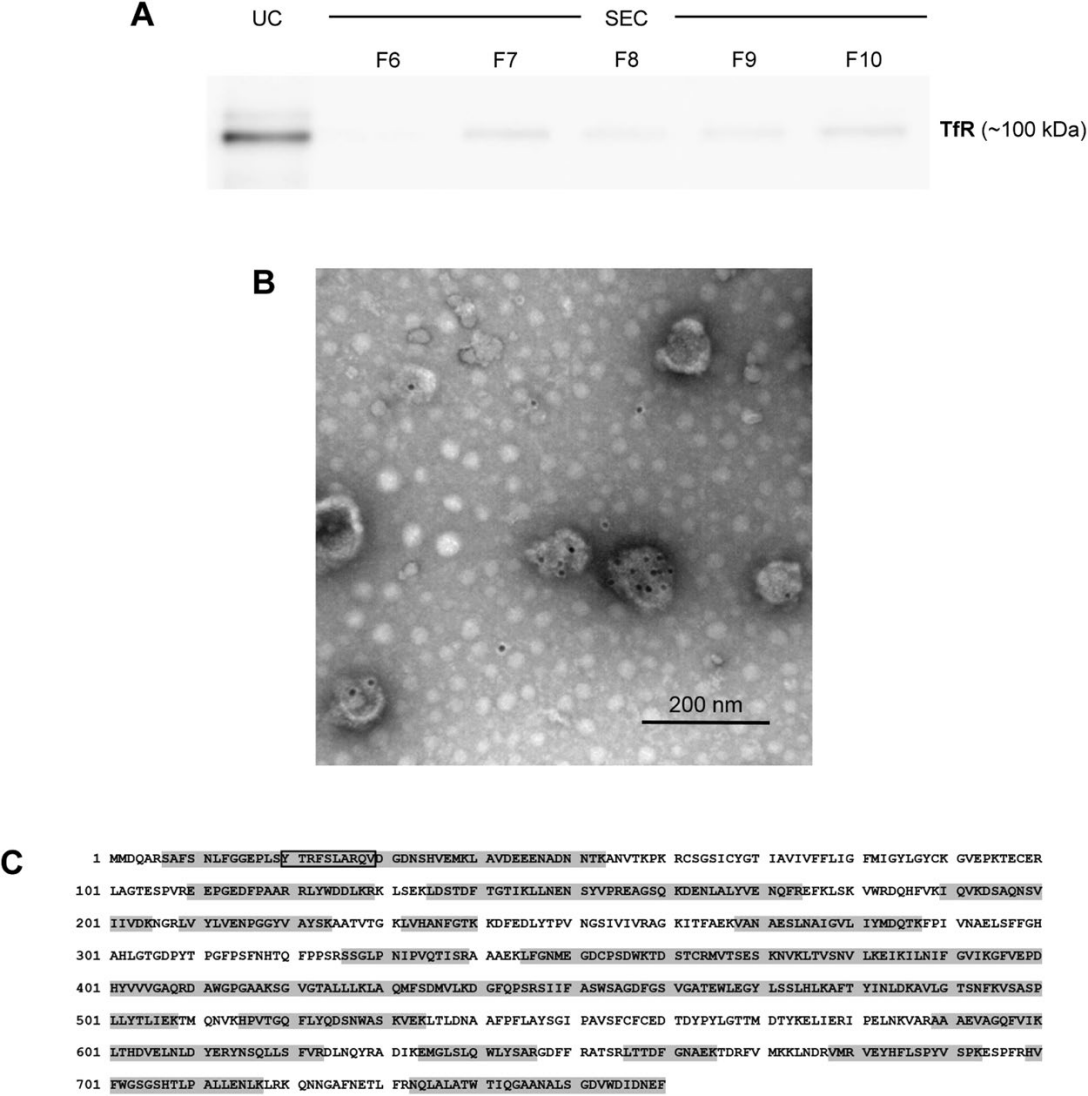
Detection of the transferrin receptor (TfR) in human reticulocyte-derived exosomes. (**A**) Immunoblot in *HuRex* purified by UC and SEC fractions. **(B)** Immunogold labelling of UC-*HuRex* using a secondary antibody conjugated to 10 nm-gold spheres. Scale bar represents 200 nm. **(C)** Protein coverage by unique peptides (grey boxes) identified by MS. The peptide sequence YTRFSLARQV, corresponding to the binding domain of TfR for hsc71^42^, is boxed in black. UniProtKB – P02786 TfR sequence is shown.

The transferrin receptor is completely and selectively removed in exosomes during the terminal differentiation of reticulocytes into erythrocytes^4,5,8^. We first analyzed the presence of this protein by immunoblot in *HuRex* purified by UC and SEC (Fig. 2A and Supplementary Fig. S2). As expected from concentration of particles/µL (Fig. 1A), a higher signal was observed in the UC preparation as compared to SEC fractions. Next, immunoelectron microscopy demonstrated that TfR is associated with exosomes corroborating their reticulocyte origin (Fig. 2B).

### Proteomic analysis of human reticulocyte-derived exosomes

**Figure 3:**
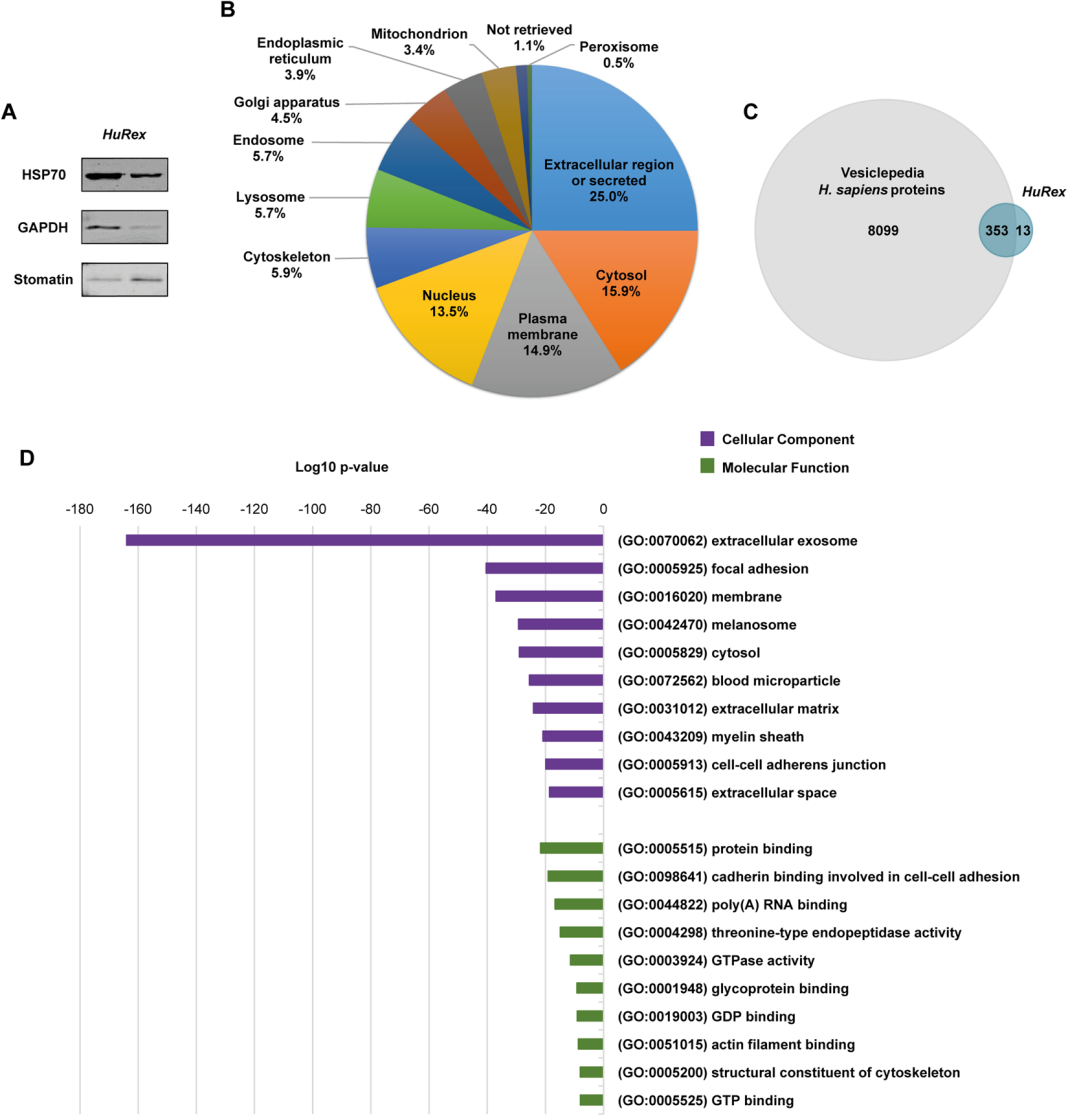
Proteome of human reticulocyte-derived exosomes identified by LC-MS/MS. (**A**) Western blot validation for HSP70, GAPDH and stomatin on UC-*HuRex*. Samples were purified from two cord blood donors, each one loaded in a different lane. **(B)** Distribution of the proteome of *HuRex* in subcellular location categories retrieved from UniProt database according to Gene Ontology (GO) annotation. **(C)** Venn diagram showing the overlap of proteins detected in *HuRex* and those reported in Vesiclepedia, a database of extracellular vesicle cargo^30^. **(D)** GO term-enrichment analysis of *HuRex* proteome at cellular component and molecular function level performed with Database for Annotation, Visualization and Integrated Discovery (David 6.8)^60^. The most over-represented GO terms are shown.

Characterization by mass spectrometry, LC-MS/MS, was performed over *HuRex* from six different cord blood donors to determine their molecular composition. As research on the molecular cargo of exosomes is largely confounded by the lack of a “gold-standard” methodology^24^, we applied several approaches for obtaining *HuRex* prior to MS. *HuRex* preparations were obtained in absence of serum (AS) from three donors and isolated by means of SEC (n = 3) and UC (n = 3). *HuRex* preparations in the presence of serum were obtained from three other donors and purified by SEC (n = 3) and UC (n = 1). In total, 367 different proteins were identified in the 10 MS-dataset according to UniProt accessions, although protein numbers from each cord blood were different from each other due to the intrinsic variability of such samples (Supplementary Table S1). Nevertheless, most of the proteins identified by SEC are a subset of the ones identified by UC (not shown). Thus, after removal of leukocytes, the use of SEC and culturing of human reticulocytes from cord blood in the presence of serum, depleted of exosomes, seems a robust method for furthering studies of *HuRex*. To further verify the reticulocyte origin of exosomes^4,5,8^, we first demonstrated the presence of TfR in all MS-datasets (Supplementary Table S1). In addition, 45 unique peptides, including the sequence YTRFSLARQV previously shown to interact with hsc70^29^, and covering 64.08% of this receptor, were identified (Fig. 2C). Further western blotting analysis on these vesicles confirmed the detection of the raft-associated protein stomatin, previously associated to *HuRex*^14^ and verified the identification of newly detected proteins such as HSP70 and GAPDH (Fig. 3A and Supplementary Fig. S2).

Proteins were assigned different subcellular locations and percentages according to Gene Ontology (GO) annotation obtained from UniProt: Extracellular region (25.0%), Cytosol (15.9%), Plasma membrane (14.9%), Nucleus (13.5%), Cytoskeleton (5.9%), Lysosome (5.7%), Endosome (5.7%), Golgi apparatus(4.5%), Endoplasmic reticulum (3.9%), Mitochondrion (3.4%), “Not retrieved” (1.1%) and Peroxisome (0.5%) (Fig. 3B and Supplementary Table S1). Of note, most of the proteins identified in this work were already listed in Vesiclepedia, a public data repository for extracellular vesicle cargo^30^ (Fig. 3C). Moreover, in agreement with these data, over-represention GO analysis of cellular component revealed that the bulk of proteins identified in *HuRex* correspond to extracellular exosome (Fig. 3D and Supplementary Table S2). According to the idea that *HuRex* remove adhesins^7,8^, GO analysis of molecular function revealed protein binding as a major function of *HuRex* (Fig. 3D and Supplementary Table S2).

Biochemical analysis of exosomes obtained from sheep reticulocytes originally demonstrated that in addition to TfR several membrane enzymes and transporters were also selectively removed by exosomes^6^. Accordingly, we identified several of such and other membrane proteins, namely, Na+/K+ transporting ATPase (ATP1A1, ATP1B3), calcium-transporting ATPase (ATP2B4), neutral amino acid transporters (SLC1A5, SLC43A1, SLC7A5), and glucose transporters 1, 2, 3 and 4 (SLC2A1, SLC2A2, SLC2A3, SLC2A4). A notable exception was acetylcholinesterase which was reported to have high activity in the original description of exosomes^6^. Other MS studies of human reticulocytes and reticulocyte-derived exosomes from other species^10,15^ have also failed to detect this enzyme, but without other alternative method confirming its absence, we cannot rule out this is due to technical issues. Several other plasma membrane proteins previously not reported in *HuRex* were identified in our analysis. These include several integrins alpha and beta (ITGA2B, ITGA4, ITGAM, ITGB1, ITGB2, ITGB3) and transporters (SLC6A9, SLC7A1) among others (Supplementary Table S1).

Previous functional enzymatic analyses of reticulocyte-derived exosomes revealed little or no activity of the cytosolic enzymes lactate dehydrogenase, glucose-6-phosphate dehydrogenase, glyceraldehyde-3-phosphate dehydrogenase, and 6-phosphogluconate dehydrogenase^6^. All these enzymes were identified in our MS analysis, *albeit* with different coverage and numbers of unique peptides (Supplementary Table S1). Of note, glyceraldehyde-3-phosphate is inactive when binding to band 3^31^ but this explanation for the lack of activity was discarded as band 3 was not acknowledged to be present in reticulocyte-derived exosomes^6^. Yet, our studies identified band 3 suggesting this alternative explanation. Other cytosolic enzymes and proteins associated with *HuRex* are worth mentioning. Several Rab GTPases were identified, in particular Rab7a, Rab11b, and Rab14. In contrast, we failed to identify Rab27, known to play a main role in exosome biogenesis in other cells^32^. Similarly to acetylcholinesterase, further experimentation would be required to demonstrate its absence or by the contrary, prove its association to *HuRex*. We also detected five S100 proteins, calcium-modulated proteins pertaining to a vertebrate multigene family which in humans contain 24 members^33^. Notoriously, we identified S100-A9 with a coverage of 89.47%, a protein that has been recently found in plasma-derived exosomes associated to chronic lymphocytic leukemia^34^. We have identified several histones, including histone H4 previously shown to be largely exported to the cytoplasm^15^. Last, we and others^15^ failed to identified Tsg101, a major player in the biogenesis of exosomes in other cells^35^.

We crossed *HuRex* proteome with reported red cell MS proteomes from human reticulocytes^15–17^ and mature RBCs^17–20^. For that we first compared the cell proteomes (Supplementary Fig. S3) and we defined a reticulocyte core proteome of 587 proteins and a mature RBC core proteome consisting of 1055 proteins. The intersection of *HuRex* proteins with the core proteomes of reticulocytes and matured RBCs (Supplementary Fig. S3 and Supplementary Table S3) showed that many of the proteins identified in *HuRex* are also detected in these works, most of them associated to plasma membrane and cytosol (Supplementary Table S3). Moreover, when we performed a direct comparison of the proteins identified in *HuRex* with the mentioned human reticulocyte proteomes^15–17^, around 70% of the proteins described in *HuRex* have been detected in their cell of origin (Supplementary Fig. S3).

Even though in this first MS proteomic description of *HuRex* we focused on protein identification, we calculated the normalized spectral abundance factor (NSAF)^36^ (one of the most common protein quantification indexes used in label-free quantificacion proteomics based on the spectral counting)^37^ to estimate relative protein abundance. We observed that previously detected proteins in *HuRex* such as TfR and stomatin, as well as newly identified like S100A9 and HSPA8 were found among the 50 most abundant proteins (Supplementary Fig. S4 and Supplementary Table S5).

### Capture of *HuRex* by dendritic cells

**Figure 4:**
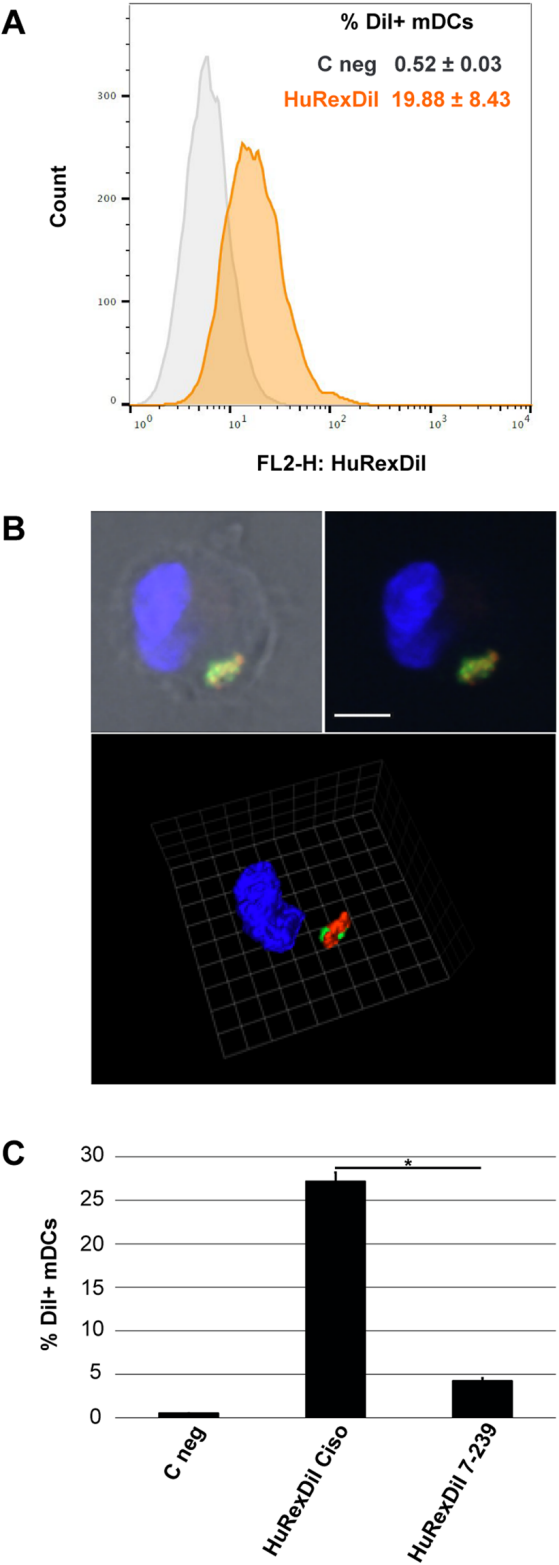
Siglec-1 dependent capture of human reticulocyte-derived exosomes by mature dendritic cells. (**A**) Flow cytometry analysis of *HuRex*_*DiI*_ capture by mDCs. **(B)** Confocal microscopy co-localization of *HuRex*_*DiI*_ (in red) and VLP_HIV-Gag-eGFP_ (in green) in mDCs (nuclei stained with DAPI). Top: z-plane showing fluorescence and bright field (scale bar 5 μm); Bottom: 3D reconstruction of z-planes (reference scale unit 2,48 μm). **(C)** Inhibition of *HuRex*_*DiI*_ capture by mDCs by blocking of Siglec-1 with α-Siglec-1 mAb. mDCs not treated with antibodies nor with *HuRex*_*DiI*_ were incubated in parallel (C neg). Data show mean values and SD from 2 donors. T-test *P* < 0.05.

MHC class-I molecules are involved in antigen presentation where professional antigen presenting cells such as dendritic cells are usually required to process and present antigens to T cells^2^. As we detected HLA class-I antigens in *HuRex* and our own previous results demonstrated the presence of MHC class-I molecules in exosomes from rodent malaria infections^11,12^, we decided to run a functional assay to determine if *HuRex* could be uptaken by dendritic cells (DCs). This assay had previously demonstrated that the sialic acid-binding Ig-like lectin 1, Siglec-1, is responsible for exosome capture on mature DCs and follows the same trafficking route as HIV-1 particles^38,39^. Flow cytometry analysis revealed that 19 ± 8% of activated monocyte-derived dendritic cells (mDCs) actively uptake *HuRex* labeled with Dil (*HuRex*_*DiI*_) (Fig. 4A). We next assessed if *HuRex*_*DiI*_ were retained within the same sub-cellular compartment as fluorescent HIV-1 VLP. As shown in Fig. 4B and Video 1, mDCs exposed to *HuRex*_*DiI*_ and subsequently exposed to HIV-1 VLP retained both types of vesicles within the same sack-like compartment, as analyzed by confocal microscopy. Last, pretreatment of mDCs with a monoclonal antibody against Siglec-1 significantly inhibited *HuRex*_*DiI*_ capture, T-test *P* < 0.05 (Fig. 4C). Thus, *HuRex* are efficiently captured by mDCs via Siglec-1.

## Discussion

**Figure 5:**
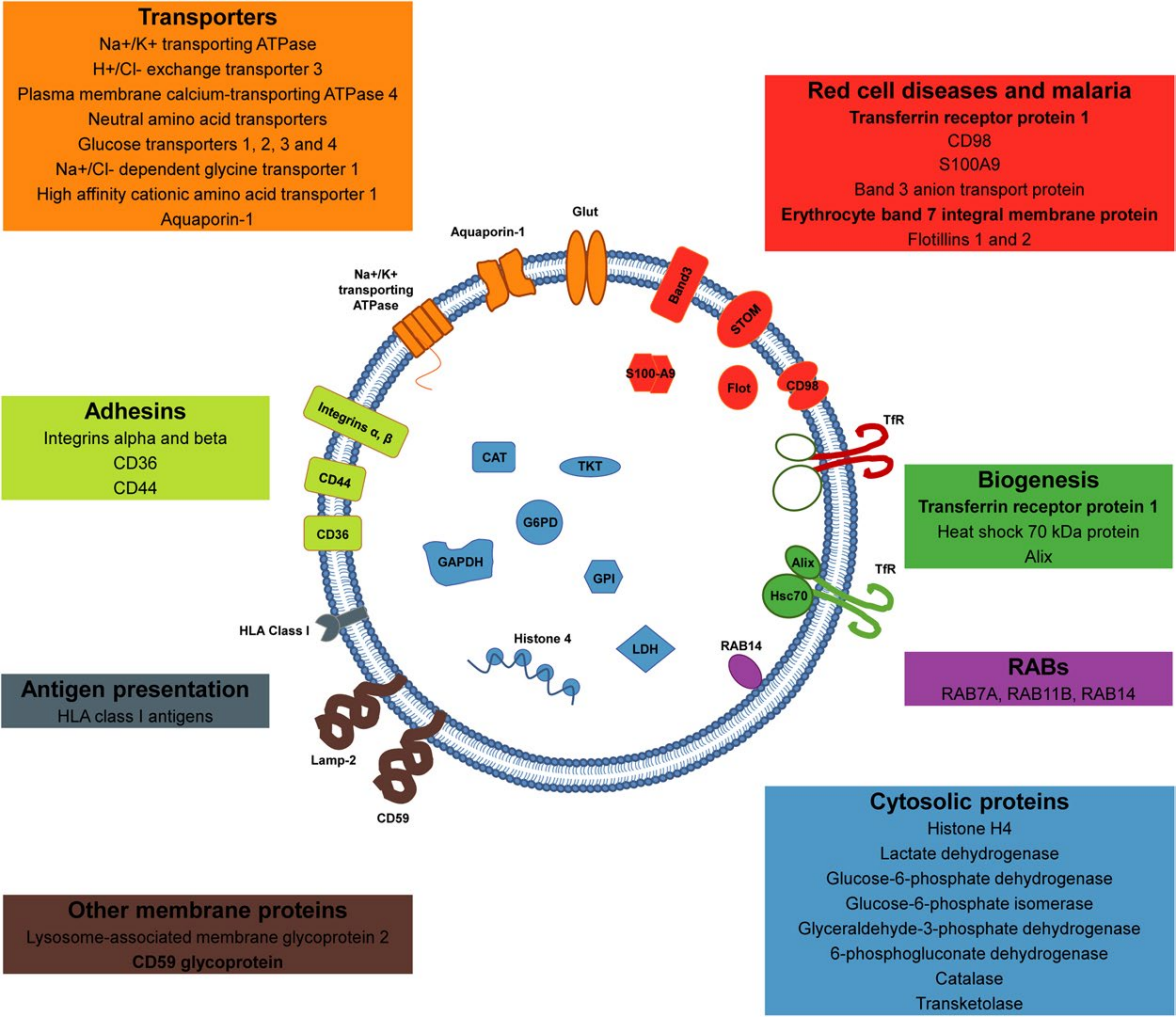
The human reticulocyte-derived exosome proteome. Schematic illustration of a human reticulocyte-derived exosome highlighting selected plasma membrane and cytosolic proteins. Transporters (in orange): Na+/K+ transporting ATPase (ATP1A1, ATP1B3), H+/Cl− exchange transporter 3 (CLCN3), Plasma membrane calcium-transporting ATPase 4 (ATP2B4), Neutral amino acid transporters (SLC1A5, SLC43A1, SLC7A5), Glucose transporters 1, 2, 3 and 4 (SLC2A1, SLC2A2, SLC2A3, SLC2A4), Na+/Cl− dependent glycine transporter 1 (SLC6A9), High affinity cationic amino acid transporter 1 (SLC7A1) and Aquaporin-1 (AQP1). Adhesins (in lime): Integrin alpha and beta (ITGA2B, ITGA4, ITGAM, ITGB1, ITGB2, ITGB3), CD36 and CD44. Other membrane proteins (in brown): Lysosome-associated membrane glycoprotein 2 (LAMP2) and CD59 glycoprotein (CD59). Antigen presentation (in grey): HLA class I antigens (HLA-A, HLA-C). RABs (in pink): RAB7A, RAB11B and RAB14. Biogenesis (in green): Transferrin receptor protein 1 (TFRC), Heat shock 70 kDa protein (HSPA8, HSPA1A) and Alix (PDCD6IP). Cytosolic proteins (in blue): Histone H4 (HIST1H4A), Lactate dehydrogenase (LDHA, LDHB), Glucose-6-phosphate dehydrogenase (G6PD), Glucose-6-phosphate isomerase (GPI), Glyceraldehyde-3-phosphate dehydrogenase (GAPDH), 6-phosphogluconate dehydrogenase (PGD), Catalase (CAT) and Transketolase (TKT). Red cell diseases and malaria (in red): Transferrin receptor protein 1 (TFRC), CD98 (SLC3A2, SLC7A5), S100A9, Band 3 anion transport protein (SLC4A1), Erythrocyte band 7 integral membrane protein (STOM) and Flotillins 1 and 2 (FLOT1, FLOT2). Proteins in bold have been previously described in human reticulocyte-derived exosomes (TfR, STOM, CD59)^7,13,14^.

Here, we present the first mass spectrometry-based proteomic analysis of human reticulocyte-derived exosomes (*HuRex*) consisting of 367 proteins. Previously, MS proteomes from human reticulocytes^15–17^, and mature RBCs^17–20^, had been reported. When comparing this novel *HuRex* proteome with those proteomes, we found many common proteins, especially from plasma membrane and cytosol (Supplementary Fig. S3 and Supplementary Table S3). These data are largely in agreement with the idea that exosomes reflect a “cell biopsy”, but at the same time are enriched in specific cargo^1,40^. In addition, the complete MS proteome of reticulocyte-derived exosomes from rats is available^10^ and its comparison with the *HuRex* proteome revealed more than 200 conserved proteins (Supplementary Table S4). This dataset thus represents the first comprehensive list of proteins from human reticulocyte-derived exosomes and a valuable resource to pursue different studies (Fig. 5).

The endocytic pathway in reticulocytes differs from other cells as lysosomes are almost completely absent. Moreover, as a Golgi apparatus and an endoplasmic reticulum are also vestigial, it is reasonable to think that complex and diverse sorting pathways are absent from reticulocytes. Thus, the final step in this differentiation should be considered the MVB-exosomal release^41^. Elegant studies on the fate of the TfR during reticulocytes maturation using rodent and sheep models demonstrated that this receptor specifically interacts with the heat shock cognate 70 kDa protein^29^. Moreover, it was later shown that the hydrophobic peptide sequence YTRFSLARQV containing basic amino acids within the cytosoloic domain of TfR represented the binding domain of hsc70^42^. However, when inhibitors of hsc70 were added to the binding assays, secretion of TfR was not affected indicating that TfR/hsc70 interactions were not the signal for sorting of TfR into exosomes. Rather, in these same studies, Alix a well-known protein involved in the biogenesis of exosomes from other cells, was also shown to interact with the YTRF motif suggesting a contribution to TfR exosomal sorting. In addition, it was shown that sorting of TfR was concomitant with proteosomal degradation of abundant cytosolic proteins via AP2 complexes.

Our MS analysis revealed the presence of TfR, including the sequence YTRFSLARQV, hsc71 protein (another name for hsc70) and Alix whereas abundant cytosolic proteins were absent. It therefore seems that similar mechanisms operate in the biogenesis and sorting of human reticulocyte-derived exosomes excepting for Tsg101 which is not present in human reticulocytes^15^ neither in our analysis. Last, Galectin 5 is a rat-specific protein involved in an ESCRT-independent sorting pathway of some glycosylated proteins^43^. We identified Galectin 3 binding protein in our dataset. Whether it has a similar function as Galectin 5 remains to be determined.

Reticulocyte-derived exosomes from experimental infections of mice with a reticulocyte-prone rodent malaria parasite contain parasite antigens and MHC class-I molecules^12^. Moreover, when used in immunizations, they were able to confer full and long-lasting protection upon lethal challenges as well as eliciting effector memory T cells^11^. Noticeably, we identified HLA class-I antigens in *HuRex* and our own unpublished results have also identified HLA class-I antigens and parasites antigens in plasma-derived exosomes from malaria patients. Elegant studies on the mechanism of antigen-specific T cell stimulation by peptide-bearing exosomes demonstrated activation of T cell proliferation mainly in the presence of mature dendritic cells^44^. We thus used a functional assay^38^ to demonstrate the potential capacity of *HuRex* to interact with dendritic cells as they play a pivotal role in antigen presentation in natural malarial human infections^45^. This assay previously demonstrated that ganglioside-containing vesicles such as viral-like particles or exosomes could be captured by mature dendritic cells expressing Siglec-1^39^. Noticeably, our results demonstrated Siglec-1-dependent specific capture of *HuRex* by mDCs as well as co-localization of *HuRex* with VLPs (Fig. 4). These data suggest that in addition of a “garbage-disposal” mechanism for the terminal differentiation of reticulocyte to erythrocytes, *HuRex* can facilitate antigen presentation via dendritic cells in the context of a human infection with a unique tropism for reticulocytes.

Studies on the role of extracellular vesicles, including exosomes, in red cell diseases are just beginning but already promise to shed light into mechanistic insights of pathology and in identifying non-invasive biomarkers^46^. For instance, in sickle cell disease (SCD), a group of inherited cell disorders, as well as in thalassemias, characterized by abnormal haemoglobin production, studies on the levels of platelets and red blood cells microparticles revealed an elevated level as opposed to normal individuals^47,48^. Moreover, a casuistic association on the abnormal presence of α4β1 integrin in circulating red blood cells leading to vascular obstruction in patients with SCD has been observed^49^. Human malaria caused by *Plasmodium vivax*, a parasite with a unique tropism for reticulocytes, is a red cell disease inducing inducing hemolytic anemia upon infections^50^. Knowledge on this parasite and disease have been hampered due to the lack of a continuous *in vitro* culture system. Our data shows that several receptors and proteins involved in entrance of the parasite into the host cell, such as CD98^51^, CD71^52^, band 3^53^ and erythrocyte band 7^54^, are selectively removed in *HuRex*. Remarkably, a recent report provides the first direct evidence of the role of exosomes in erythroid diseases. It was shown that the S100-A9 protein associated to exosomes represent a new pathway for NF-κβ activation in chronic lymphocytic leukemia cells^34^. In addition, extracellular vesicles in hematological malignancies have been stood out not only as promoters of tumor aggressiveness, but also as a valuable source of biomarkers for diagnosis^55^. Further studies on extracellular vesicles, including *HuRex*, in the context of red cell diseases are warranted.

In conclusion, this work opens new avenues for furthering studies of human reticulocyte-derived exosomes: firstly, on their biogenesis as most of the data presently available on this subject are from other species; secondly, on the possibility that *HuRex* can present pathogen-peptide antiges via dendritic cells inducing T cell responses; thirdly, on guiding new approaches to develop a continuous *in vitro* culture system for *P. vivax*; fourthly, on comparative analysis of the molecular composition of reticulocyte-derived exosomes from plasma or other body fluids in patients with difficulty to diagnose red cell diseases thus providing an alternative for identifying novel biomarkers. Last but not least, as quoted elsewhere^8^, perhaps Rose Johnstone was right: after having been a curiosity for many years, we might be at the doorstep of recognizing the potential of reticulocyte-derived exosomes.

## Materials and Methods

### Purification of human reticulocytes

Human cord blood samples were obtained from the Blood and Tissue Bank of Barcelona (https://www.bancsang.net/) in accordance with the Good Clinical and Laboratory Practice Guidelines, the Declaration of Helsinki, and local rules and national regulations. The protocol, including the informed consent forms, has been approved by the Clinical Research Ethics Committee of Vall d’Hebron University Hospital (PR(CS)236/2017). Briefly, similarly to a previous work^56^, 70 mL of cord blood were centrifuged at 1000 g for 15 min. After removal of plasma, the pelleted cells were washed and re-suspended at 50% hematocrit with RPMI-1640 medium (Sigma). Leukocytes and platelets were removed by filtration through columns of cellulose powder (Whatman), previously packed with 10 mL of cellulose, irradiated with ultraviolet light for sterilization and washed with a double volume of RPMI medium immediately before their use. Filtrated blood was washed twice with RPMI medium and adjusted to 50% hematocrit. 5 mL aliquots of blood were carefully layered on 6 mL 70% Percoll (Healthcare). After centrifugation at 1200 g for 15 min at 20 °C, concentrated reticulocytes in the Percoll interface were carefully collected and washed twice with RPMI medium. Reticulocyte quantification was performed by examination of brilliant cresyl blue (BCB)/Giemsa stained thin blood films^57^. Only samples with >20% reticulocyte-enrichments and <2% leukocytes were used for *HuRex* production. We routinely obtained in the order of 10^8^ reticulocytes per 70 mL of cord blood.

### *In vitro* production and isolation of human reticulocyte-derived exosomes

Reticulocytes were cultured for 36 h at 37 °C and 1–3% hematocrit in RPMI medium, not supplemented or supplemented with 0.5% of human AB serum, previously depleted of exogenous exosomes. Viability of reticulocytes was assessed using Trypan Blue stain 0.4% (Sigma, 93595). Cell-free post-culture supernatants were collected by centrifugation at 1,300 g for 20 min at 20 °C. To isolate Rex, 2 mL of culture supernatants were loaded on 10 mL-sepharose CL-2B (Sigma) size-exclusion chromatography (SEC) columns^26^, where fractions of 500 µL were collected. Alternatively, 6–10 mL of culture supernatants were subjected to sequential centrifugation at 4 °C for 15,000 g/45 min and UC at 100,000 g/90 min^25^, where exosome pellets were resuspended in 200 µL of PBS. Protein content of SEC fractions and UC samples was determined by Nanodrop^®^ ND-1000 and BCA assay (Thermo Scientific).

### Nanoparticle tracking analysis (NTA)

Size distribution and concentration of exosomes were determined by NTA using a NanoSight LM10 instrument (Malvern Instruments Ltd) as described^26^. Data were analyzed with NTA software (version 3.1).

### Bead-based flow cytometry

SEC fractions enriched in exosomes were identified by bead-based flow cytometry^25,26^ assessing the presence of the transferrin receptor, CD71, a major component of *Rex*^4,5,8^. UC samples were similarly analyzed to warrant the presence of this marker. Briefly, SEC fractions and UC samples were coupled to 4 µm-aldehyde/sulfate-latex beads (Invitrogen) for 15 min at RT. Beads were then resuspended in 1 mL of bead-coupling buffer (BCB: PBS with 0.1% BSA and 0.01% NaN_3_) and incubated overnight at RT on rotation. Exosome-coated beads were then centrifuged at 2,000 g for 10 min at RT, and washed once with BCB prior to incubation with anti-CD71 (ab84036) at 1:1,000 dilution for 30 min at 4 °C. After washing with BCB, exosome-coated beads were incubated for 30 min at 4 °C with Alexa Fluor 488-conjugated anti-rabbit IgG secondary antibody (Invitrogen, A-11008) at 1:100 dilution. Coated beads incubated with secondary antibodies were used as a control. Labelled exosome-beads were washed twice with BCB before being finally resuspended in PBS and subjected to flow cytometry (FacsVerse; BD). Flow Jo software was used to compare mean fluorescence intensity (MFI) of exosome-coated beads.

### Immunoblot analysis

Ten µL aliquots of either a UC preparation or individual SEC fractions were analyzed by Western blot against human CD71. Membranes were probed with rabbit polyclonal anti-CD71 (ab84036) at 1:250 dilution for 1 hour at RT. A goat anti-rabbit IgG coupled to HRP (Sigma, A6154) was used at a dilution of 1:2,500 for 1 hour at RT. Revealing was performed using ECL Western Blotting Substrate (Pierce™) in ImageQuant LAS 4000 (GE Healthcare Life Sciences). Additionally, 20 µL aliquots of UC preparations were analyzed to confirm the presence of HSP70, GAPDH and stomatin in *HuRex*. Membranes were incubated for 1 hour at RT with primary antibodies anti-HSP70 (Santa Cruz Biotechnology, W27 sc-24) at 1:250 dilution, anti-GAPDH (Sigma, G9545) at 1:500 dilution or anti-stomatin (Invitrogen, PA5-30019) at 1:250 dilution. Subsequently, membranes were washed and incubated for 1 h at RT with the Li-Cor IRDye-labeled secondary antibodies IRDye® 800CW goat-anti-mouse (925-32210, Li-Cor Biosciences) at 1:15,000 dilution or IRDye® 680RD goat anti-rabbit (925-68021, Li-Cor Biosciences) at 1:20,000 dilution. Blots were analyzed with the Odyssey near-infrared system (Li-Cor Biosciences) having the intensity of 700 channel set up at 5 and the one of 800 channel at 7. Images were edited using the software Image J (NIH).

### Transmission electron microscopy (TEM)

TEM was performed over *HuRex* UC preparations negatively stained as previously detailed^12^. Preparations were analyzed with a Jeol JEM-1100 TEM (Jeol Ltd, Tokyo, Japan) equipped with a Gatan Ultrascan ES1000 CCD Camera. Immunogold labeling was performed as detailed elsewhere^26^ with anti-CD71 (ab84036).

### *HuRex*_*DiI*_ generation

Reticulocyte-enriched RBCs were labeled with DiI (Molecular Probes, V-22885) following manufacturer’s instructions. Culture of stained cells and exosome isolation was performed as for the unstained *HuRex*. Fluorescence of *HuRex*_*DiI*_ was determined by a Fluoroskan Ascent FL fluorimeter (Thermo Fisher Scientific).

### Exosome and VLP capture by mature monocyte-derived DCs via Siglec-1

2 × 10^5^ LPS-matured monocyte-derived DCs (mDCs) were obtained as previously described^38^ and incubated with 50 μg *HuRex*_*DiI*_ at 37 °C for 4 hours. Cells were washed and acquired with a BD FACSCalibur™ cytometer. Data were analyzed using FlowJo software. For microscopy analysis, mDCs were pulsed for 24 h with *HuRex*_*DiI*_ and then with 150 μg of fluorescent viral-like particles containing Gag-enhanced green fluorescent (VLP_HIV-Gag-eGFP_) for 3 additional hours at 37 °C. Cells were washed, fixed, cytospun into coverslips, mounted with DAPI mounting media (Thermo Fisher Scientific) and analyzed with an Ultraview ERS Spinning Disk System (Perkin-Elmer) mounted on a Zeiss Axiovert 200 M inverted microscope. Volocity software (Perkin-Elmer) was used to analyze microscopy images.

To determine whether Siglec-1 could represent a receptor for entrance of *HuRex*, mDCs were pre-incubated with 10 µg/mL of an anti-Siglec-1 monoclonal antibody (clone 7–239; Abcam) or a mouse anti-human IgG1 isotype control (BD) at RT for 15 min^39^ before addition of 50 μg *HuRex*_*DiI*_ at 37 °C for 4 hours. Capture was analyzed by flow cytometry as described above.

### Mass spectrometry

Liquid chromatography (nanoLCULTRA-EKSIGENT) followed by mass spectrometry (LC-MS/MS) on a LTQ Orbitrap Velos (Thermo Fisher Scientific) was performed following our own procedures^26^. To increase exosomal protein coverage, some samples (*CB30_PS_SEC; CB30_PS_UC; CB31_AS_SEC; CB31_AS_UC*) were extracted with RIPA buffer and digested with Lys-C in addition to Trypsin digestions.

### Protein identification and *in silico* analysis

Mass spectrometry data were processed by Mascot v2.5.1 (Matrix Science) using the human sequences from the UniProt-Swiss-prot database (release April 2017) with a false-discovery rate below 1%. Proteins identified by a unique peptide were only accepted if present in two or more samples. Keratins and keratin-associated proteins as well as potential serum contaminants were removed from the final list of proteins (Supplementary Table S1). We considered as serum contaminants the classical plasma proteins described by Anderson and Anderson proteomics^58^ as well as other protein components compiled in this work that were exclusively detected in *HuRex* preparations from serum-supplemented cultures. The MS proteomics data have been deposited to the ProteomeXchange Consortium via the PRIDE partner repository with the dataset identifier PXD008545^59^.

We estimated relative protein abundance calculating the normalized spectral abundance factor (NSAF) as described elsewhere^36^. This protein quantification index was established considering all proteins identified prior to filtering with their corresponding number of peptide spectrum matches.

## Electronic supplementary material

Supplementary Information

Supplementary Table S1

Supplementary Table S2

Supplementary Table S3

Supplementary Table S4

Supplementary Table S5

Video 1
