# Supplementary material for: Proteomics study of human cord blood reticulocyte-derived exosomes
Source: Sci Rep. 2018 Sep 19;8:14046. doi: 10.1038/s41598-018-32386-2 (PMC6145868; doi:10.1038/s41598-018-32386-2)

Supplementary Information

Proteomics study of human cord blood reticulocyte-derived exosomes

Míriam Díaz-Varela,[1] Armando de Menezes-Neto,[1†] Daniel Perez-Zsolt,[2] Ana Gámez-Valero,[1††] Joan Seguí-Barber,[1] Nuria Izquierdo-Useros,[2] Javier Martinez-Picado,[2,3,4] Carmen Fernández-Becerra,[1,5*] and Hernando A. del Portillo[1,4,5*]

[1]ISGlobal, Hospital Clínic - Universitat de Barcelona, Barcelona, Spain; [2]IrsiCaixa AIDS Research Institute, Badalona, Spain; [3]Universitat de Vic – Universitat Central de Catalunya (UVic-UCC), Vic, Spain; [4]Institució Catalana de Recerca i Estudis Avançats (ICREA), Barcelona, Spain; [5]IGTP Institut d'Investigació Germans Trias i Pujol, Badalona, Spain.

[†]Current address: Instituto Aggeu Magalhães-FIOCRUZ, Recife, Pernambuco, Brazil.

[††]Current address: Department of Pathology & REMAR-IVECAT Group, Hospital Universitari and Health Sciences Research Institute Germans Trias i Pujol, Universitat Autònoma de Barcelona, Spain.

***Corresponding authors:**

Carmen Fernández-Becerra; Ctra. de Can Ruti. Camí de les Escoles, s/n, 08916 Badalona (Barcelona), Spain; carmen.fernandez@isglobal.org; Tel. (+34) 934978693

Hernando A. del Portillo; Ctra. de Can Ruti. Camí de les Escoles, s/n, 08916 Badalona (Barcelona), Spain; hernandoa.delportillo@isglobal.org; Tel. (+34) 690737229.

A. Supplementary Figures

Supplementary Figure S1. Enrichment of human reticulocyte from cord blood and isolation of reticulocyte-derived exosomes. (A) Schematic diagram of the purification of human reticulocytes and the different ways of *in vitro* production and isolation of *HuRex* assessed. Brilliant cresyl blue-stained thin smears of human cord blood **(B)** before and **(C)** after reticulocyte enrichment. Diagram was designed by the author MDV using icons from www.flaticon.com made by Freepik, Prosymbols and Vectors Market.

Supplementary Figure S2. Immunoblot analysis in *HuRex*. Full-length blots against transferrin receptor (TfR), HSP70, GAPDH and stomatin displayed in the main figures.

Supplementary Figure S3. Comparative analysis with human red cell MS proteomes. (A) Intersection of human reticulocyte MS proteomes[15–17]. Core proteome consists of 587 proteins marked in blue. **(B)** Intersection of human mature RBC MS proteomes[17–20]. Core proteome consists of 1055 proteins marked in red. **(C)** Intersection of *HuRex* proteome with the reticulocyte core proteome and the mature RBC core proteome. Those proteins common to both core proteomes (white), to reticulocytes (cyan), to mature RBCs (yellow) and those exclusively related to *HuRex* are listed in the Supplementary Table S3. **(D)** Intersection of HuRex proteome with human reticulocyte MS proteomes[15–17]. All comparisons have been performed using gene name annotation.

Supplementary Figure S4. The 50 most abundant proteins in *HuRex* by NSAF. Proteins, annotated by Gene ID, with the highest NSAF[37] median as presented in the Supplementary Table S5.

B. Supplementary Tables

Supplementary Table S1. List of proteins identified in human reticulocyte-derived exosomes from different preparations by LC-MS/MS. List of grouped proteins that have been identified at 1 %FDR and by at least 2 unique peptides, or with 1 unique peptide if they are present in two or more preparations. After filtering out contaminant proteins, a list of 367 proteins has being generated (presented in Sheet 1 "*HuRex* proteome"). Proteins are listed based on their number of unique peptides. Sheets from 2 to 13, contain the lists of proteins identified for different subcellular locations according to GO annotation: "2. ER or secreted", "3.Cytosol", "4.Plasma membrane", "5.Nucleus", "6.Cytoskeleton", "7.Lysosome", "8.Endosome", "9. Golgi apparatus", "10. Endoplasmic reticulum", "11.Mitochondrion", "12.Peroxisome", "13.Not retrieved". Sheet 14 shows the list of potential contaminants removed from the final list. Table abbreviations: ER: extracellular region.

Supplementary Table S2. Gene Ontology enrichment analysis of human reticulocyte-derived exosomes. GO term-enrichment analysis of *HuRex* proteome at biological process (Sheet 1), cellular component (Sheet 2) and molecular function (Sheet 3) level performed with **D**atabase for **A**nnotation, **V**isualization and **I**ntegrated **D**iscovery (David 6.8)[61]. All GO terms with their statistical analysis are shown.

Supplementary Table S3. Lists of proteins from the comparative analysis with human red cell MS proteomes. In sheet 1: list of proteins common to both reticulocyte and mature RBC core proteomes (white), to reticulocytes (cyan), to mature RBCs (yellow) and those exclusively related to *HuRex* (green) that were obtained according to the analysis shown in Supplementary Figure S2. In sheet 2: plasma membrane proteins and in sheet 3: cytosol proteins.

Supplementary Table S4. *Rex* proteins conserved among different species. Proteins common between *HuRex* and previously reported proteome from rat exosomes at day 2 of in *vitro* differentiation[10] (sheet 1).

Supplementary Table S5. Relative protein abundance in *HuRex* by NSAF. Relative protein abundance was estimated with the calculation of the protein quantification index NSAF[37] for every protein, denoted by each gene ID, identified in every *HuRex* preparation. Mean, median and standard deviation of the NSAF values from the all preparations are included in the last three columns (sheet 1).

C. Video 1

Confocal microscopy analysis of a LPS mDC pulsed with $HuRex_{DiI}$ and then with green fluorescent HIV-1 VLP. Movie shows 3D reconstruction of the maximum intensity fluorescence of the x-y sections collected throughout the whole cell z-volume every 0.2 µm. DAPI-stained nucleus is depicted.

Supplementary Figure S1

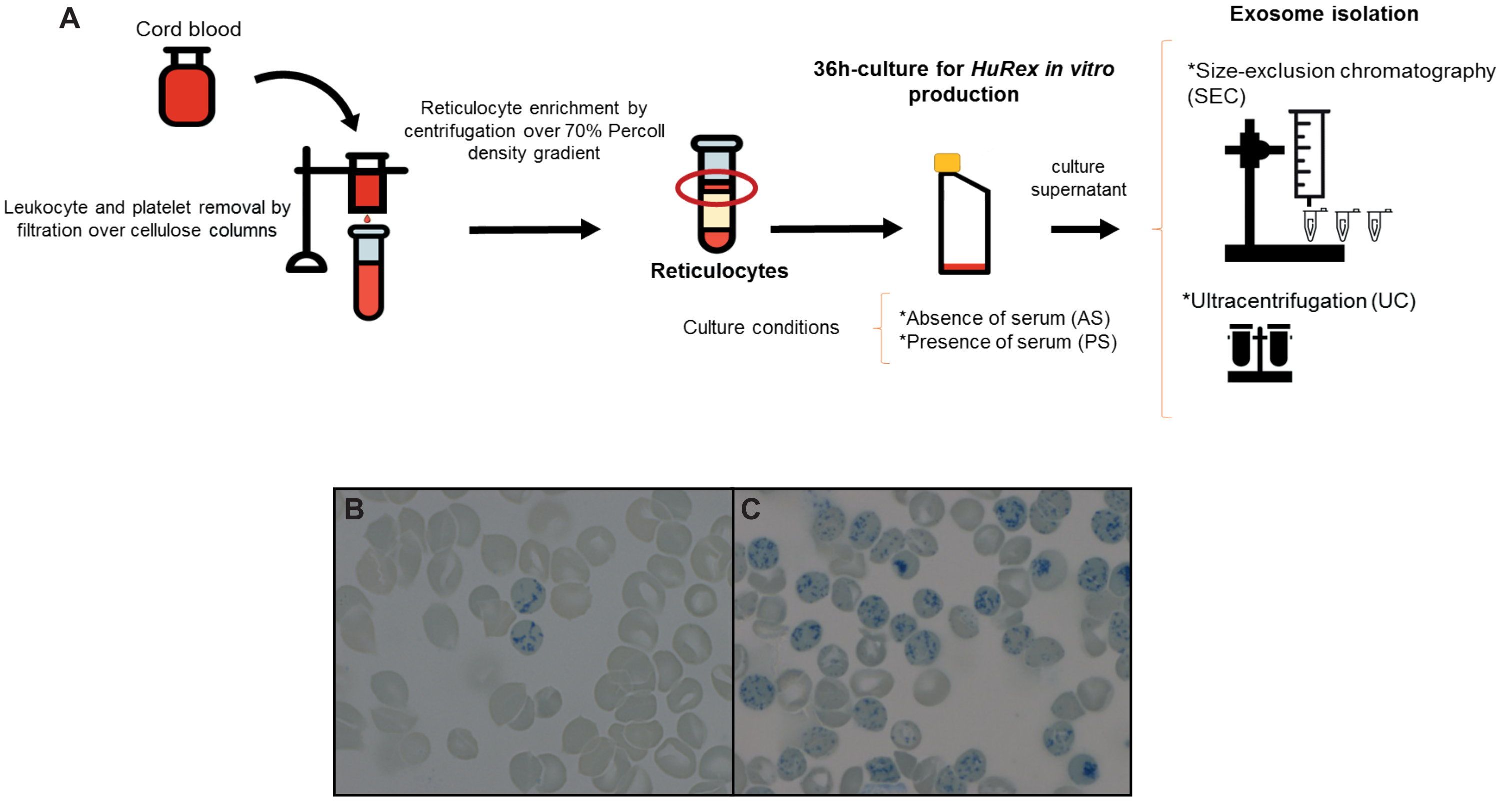

Supplementary Figure S2

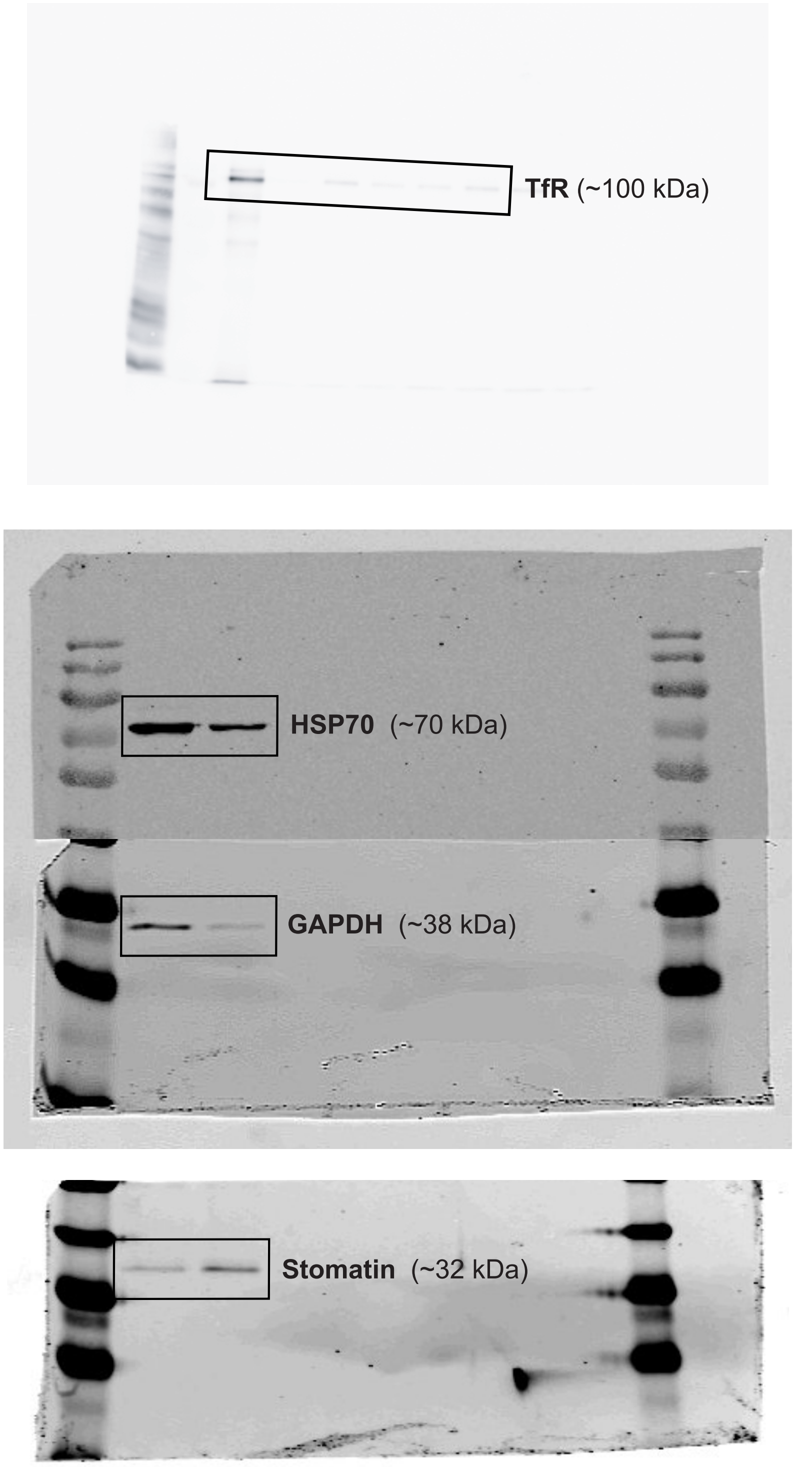

Supplementary Figure S3

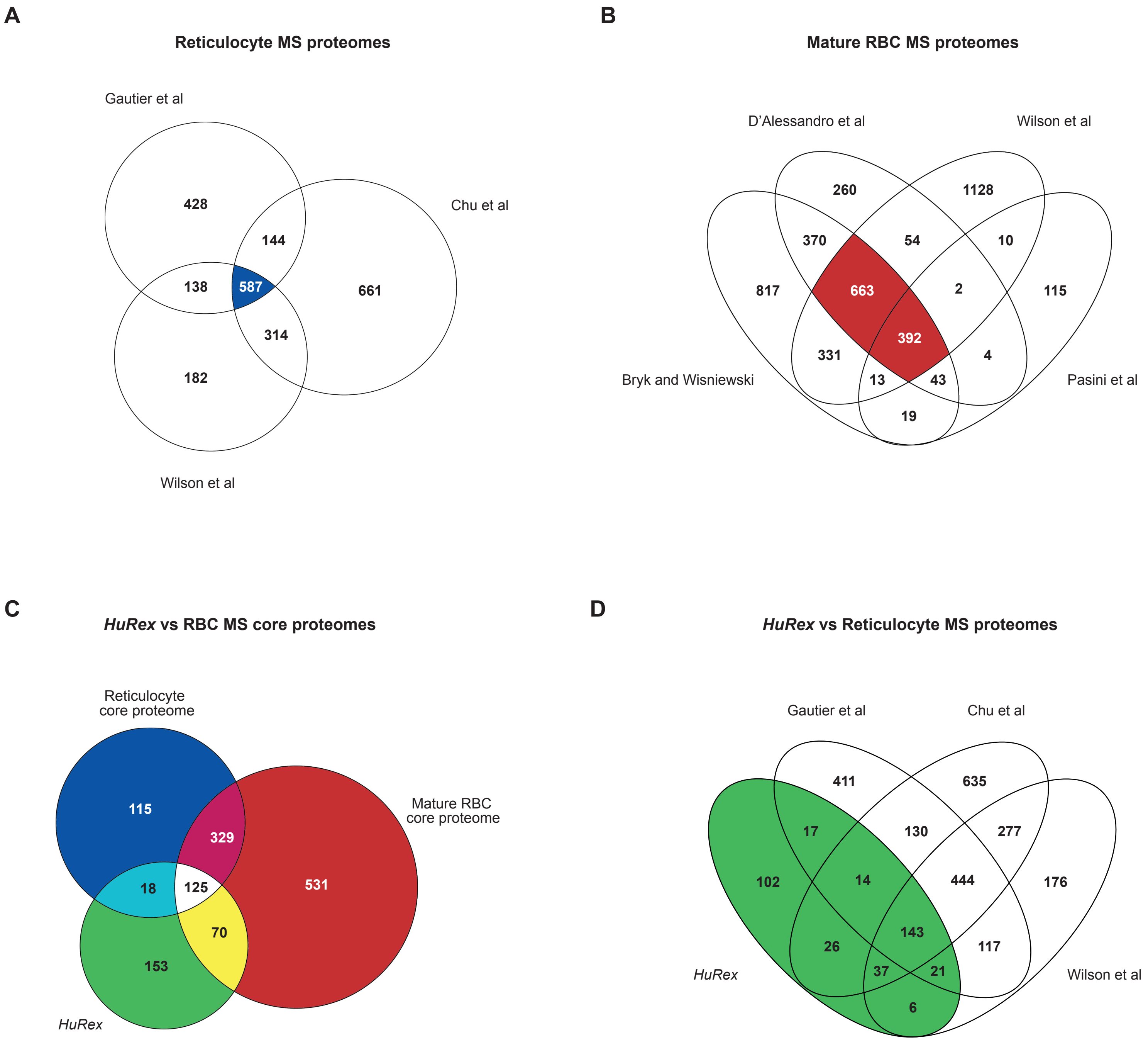

Supplementary Figure S4

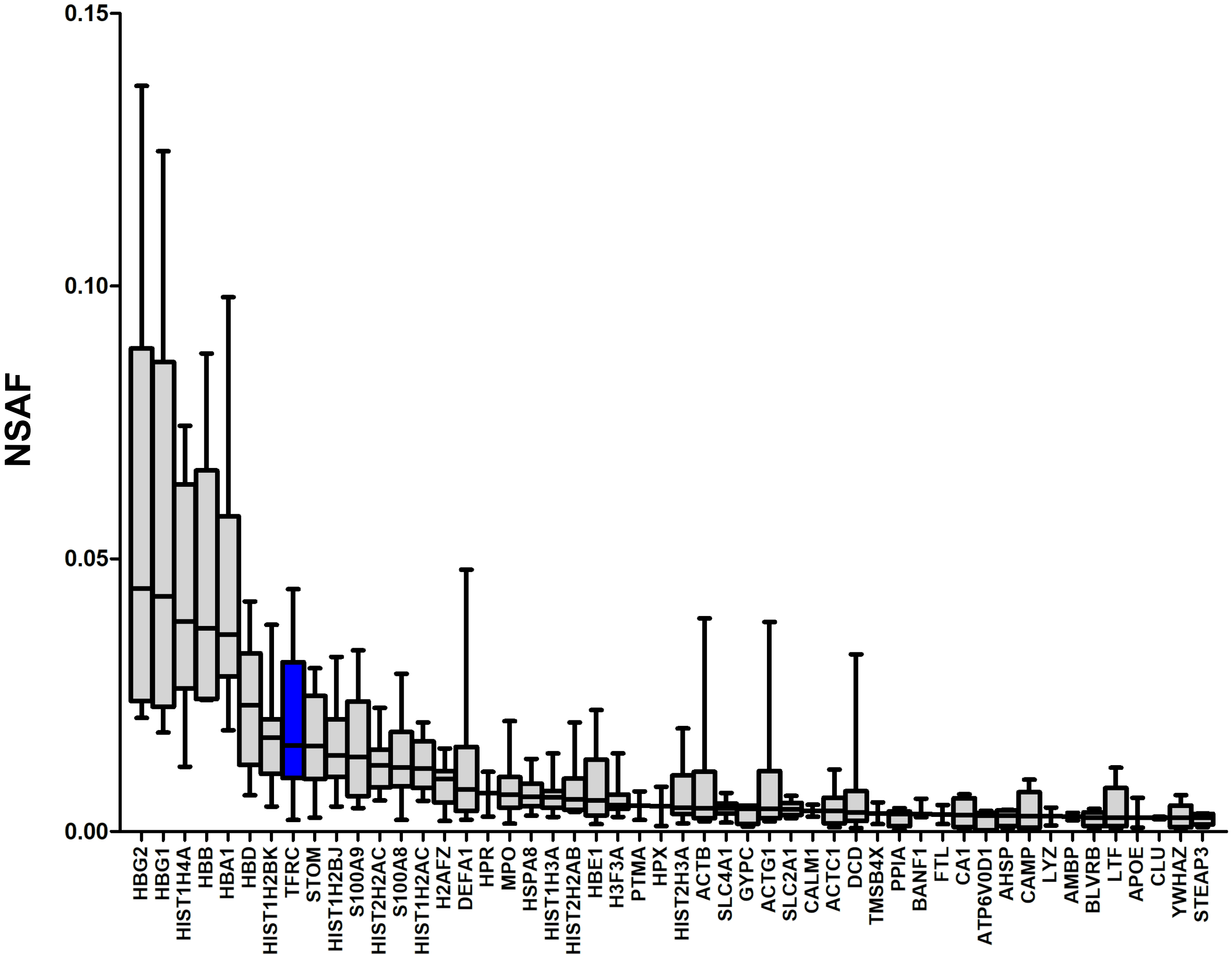

Supplement: Supplementary file 1 — Supplementary Information [file 41598_2018_32386_MOESM1_ESM.pdf]
